# Acquired resistance to DZNep-mediated apoptosis is associated with copy number gains of AHCY in a B-cell lymphoma model

**DOI:** 10.1186/s12885-020-06937-8

**Published:** 2020-05-14

**Authors:** Chidimma Agatha Akpa, Karsten Kleo, Elisabeth Oker, Nancy Tomaszewski, Clemens Messerschmidt, Cristina López, Rabea Wagener, Kathrin Oehl-Huber, Katja Dettmer, Anne Schoeler, Dido Lenze, Peter J. Oefner, Dieter Beule, Reiner Siebert, David Capper, Lora Dimitrova, Michael Hummel

**Affiliations:** 1 Department of Experimental Hematopathology, Institute of Pathology, Charité Medical University, Berlin, Charitéplatz 1, 10117 Berlin, Germany; 2 Berlin School of Integrative Oncology, Charité - Medical University of Berlin, Berlin, Germany; 3 Berlin Institute of Health, Charité Core Unit Bioinformatics, Berlin, Germany; 4 Institute of Human Genetics, Ulm University and Ulm University Medical Center, Ulm, Germany; 5 Institute of Functional Genomics, University of Regensburg, Regensburg, Germany; 6 Department of Neuropathology, Charité, Medical University of Berlin, corporate member of Free University Berlin, Humboldt-University Berlin, and Berlin Institute of Health, Berlin, Germany; 7 German Cancer Consortium (DKTK); Partner Site Berlin, German Cancer Research Center (DKFZ), Heidelberg, Germany

**Keywords:** 3- Deazaneplanocin a (DZNep), B-cell lymphoma, Enhancer of zeste homolog 2 (EZH2), S-adenosyl-L-homocysteine hydrolase (AHCY)

## Abstract

**Background:**

Enhancer of zeste homolog 2 (EZH2) is considered an important driver of tumor development and progression by its histone modifying capabilities. Inhibition of EZH2 activity is thought to be a potent treatment option for eligible cancer patients with an aberrant EZH2 expression profile, thus the indirect EZH2 inhibitor 3-Deazaneplanocin A (DZNep) is currently under evaluation for its clinical utility. Although DZNep blocks proliferation and induces apoptosis in different tumor types including lymphomas, acquired resistance to DZNep may limit its clinical application.

**Methods:**

To investigate possible mechanisms of acquired DZNep resistance in B-cell lymphomas, we generated a DZNep-resistant clone from a previously DZNep-sensitive B-cell lymphoma cell line by long-term treatment with increasing concentrations of DZNep (ranging from 200 to 2000 nM) and compared the molecular profiles of resistant and wild-type clones. This comparison was done using molecular techniques such as flow cytometry, copy number variation assay (OncoScan and TaqMan assays), fluorescence in situ hybridization, Western blot, immunohistochemistry and metabolomics analysis.

**Results:**

Whole exome sequencing did not indicate the acquisition of biologically meaningful single nucleotide variants. Analysis of copy number alterations, however, demonstrated among other acquired imbalances an amplification (about 30 times) of the S-adenosyl-L-homocysteine hydrolase (*AHCY*) gene in the resistant clone. AHCY is a direct target of DZNep and is critically involved in the biological methylation process, where it catalyzes the reversible hydrolysis of S-adenosyl-L-homocysteine to L-homocysteine and adenosine. The amplification of the *AHCY* gene is paralleled by strong overexpression of AHCY at both the transcriptional and protein level, and persists upon culturing the resistant clone in a DZNep-free medium.

**Conclusions:**

This study reveals one possible molecular mechanism how B-cell lymphomas can acquire resistance to DZNep, and proposes AHCY as a potential biomarker for investigation during the administration of EZH2-targeted therapy with DZNep.

## Background

The development of drug resistance to cancer chemotherapeutics remains a major concern in most treatment regimens. Several epigenetic-based therapies are under investigation or being employed for treatment of patients with lymphomas of B-cell origin. This is due to the important role that epigenetic alterations play in promoting tumor development and progression via downregulation of tumor suppressor genes [1]. These epigenetic modifications may involve covalent post-translational modifications at the N-termini of histones or changes in the methylation pattern of cytosine bases within the DNA, especially at CpG sites [2]. Histones are important structural components of the cell that package and organize DNA into nucleosomal units and various post-translational modifications of histones are known to contribute to transcriptional gene activity in conjunction with other mechanisms [3–8].

Enhancer of zeste homolog 2 (EZH2) is a histone methyltransferase that is involved in cellular differentiation and development in both health and disease. EZH2 promotes transcriptional repression by catalyzing the trimethylation of lysine 27 on histone 3 (H3K27me3) - a repressive histone mark. In lymphoma and other malignancies, *EZH2* gain-of-function mutations and overexpression are considered important drivers of oncogenesis because of their role in silencing tumor suppressor genes regulating apoptosis, cell cycle regulation, proliferation, migration and differentiation [9–14]. Due to its oncogenic role, the targeting of EZH2 might be a promising approach for lymphoma therapy. 3-Deazaneplanocin A (DZNep) is an indirect inhibitor of EZH2 currently in the pre-clinical phase of drug development and has been shown to promote apoptosis in various primary tumor cells and cancer cell lines [15–20]. The apoptotic effects mediated by DZNep application are more pronounced in cancer cells, with minimal effects on normal cells, and are fostered by the inhibition of the repressive H3K27me3 mark [15, 18, 21].

DZNep directly inhibits the enzyme S-adenosyl-L-homocysteine hydrolase (AHCY) that catalyzes the reversible hydrolysis of S-adenosyl-L-homocysteine (SAH) to L-homocysteine and adenosine. The direct inhibition of AHCY by DZNep leads to the build-up of the substrate SAH, which in turn causes a negative feedback inhibition of methyltransferases such as EZH2 [22]. Proper functioning of AHCY is essential for the efficient maintenance of histone methylation levels in the cell [23]. Alterations in AHCY function have been linked to cancer with varying outcomes depending on the cancer entity involved. For example, with lowered AHCY activity, the invasiveness of breast cancer and glioblastoma cell lines decreases [24, 25]. Furthermore, in hepatocellular carcinoma cells, reduced AHCY activity is associated with cell cycle inhibition and a lowered proliferation rate [23]. In esophageal squamous cell carcinoma, however, elevated AHCY levels had no effect on cell proliferation but promoted apoptosis and inhibited cell migration and adhesion [26]. Besides, aberrant AHCY expression has been observed with the transformation of follicular lymphoma to diffuse large B-cell lymphoma [27].

In this study, we investigated the underlying molecular mechanism of resistance of a B-cell lymphoma model to DZNep using a DZNep-resistant clone generated from a DZNep-sensitive cell line. We identified *AHCY* as a potential biomarker that could be of predictive relevance for therapeutic inhibition of EZH2 using DZNep.

## Methods

### Drug, cell lines and culture conditions

DZNep (Selleckchem, Germany) was dissolved in sterile water following the manufacturer’s recommendation as previously described [20].

The sporadic Burkitt lymphoma cell line BLUE-1 (ACC-594, from German Collection of Microorganisms and Cell Cultures (DSMZ) Germany) was cultured in RPMI 1640 (ThermoFisher Scientific, Germany) medium enriched with 20% fetal calf serum (PAN-Biotech, Germany). Cell lines were tested and confirmed mycoplasma negative with the MycoAlert Mycoplasma Detection kit (Lonza, Germany). All cell lines were incubated at 37 °C at 5% CO_2_.

**Fig. 1:**
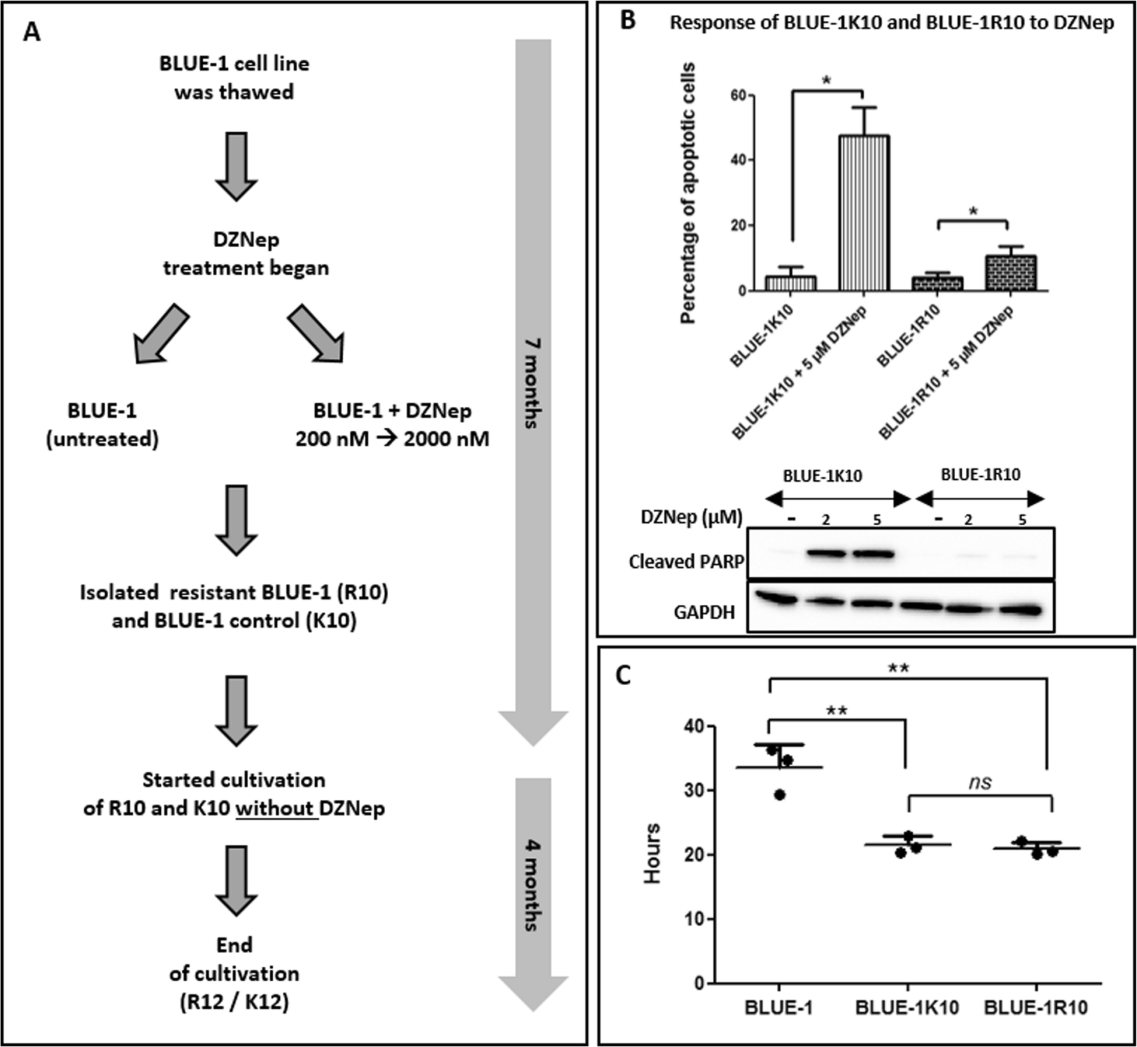
Generation and characterization of a DZNep-resistant clone. **a** Scheme of the generation of the DZNep-resistant clone and its control. **b** Comparison of the apoptotic response of BLUE-1 K10 (control) and BLUE-1R10 to DZNep. Above: The cell lines BLUE-1R10 and BLUE-1 K10 were either treated with 5 μM DZNep or untreated for 72 h. Cells were harvested and the percentage of apoptotic cells was determined by flow cytometry. Data is shown as mean plus standard deviation (SD) of three biological replicates. Below: Western blot analysis was performed using total protein lysates from both cell lines either untreated or treated with 2 μM and 5 μM DZNep, respectively. GAPDH was used as a loading control for the Western blot. The full-length blot is presented in Additional file 6: Fig. S5. The FUSION-CAP Software was used for Western blot image analysis. **c** Comparison of the doubling time of BLUE-1, BLUE-1 K10 and BLUE-1R10. The three cell lines were cultivated at a seeding density of 2 × 10^5^ cells in 6-well culture plates. The number of vital cells were measured at 24 h, 48 h and 72 h by flow cytometry. Doubling time is shown as mean plus SD of triplicate measurements. *ns*: not significant

Generation of a DZNep resistant clone was achieved by splitting the BLUE-1 culture into a control group and a treatment group (Fig. 1a). The treated group received increasing concentrations of DZNep starting from 200 nM up to 2000 nM over a period of 7 months. The cells were split 3 times a week and fresh medium without or with DZNep was added to the control and treated cells, respectively. Vital cells were counted each time by flow cytometry before the cells were split. Cryostocks were made every third to fourth week from both cell cultures. At 7 months, DZNep pressure on cultures of the treated group was removed by growing both untreated (BLUE-1 K10) and treated (BLUE-1R10) cells in medium without DZNep. About 4 months later, both cell cultures were harvested (BLUE-1 K12 and BLUE-1R12) and frozen. Prior to further use, frozen cells were thawed and maintained in a DZNep-free medium for at least 1 week.

### Flow cytometry

The BD Accuri C6 flow cytometer (Becton Dickinson Biosciences, USA) was used for the measurement of apoptotic cells and determination of the doubling time of cells. Measurement of apoptosis was performed after staining 3 × 10^5^ cells from the cell cultures with a mixture of Annexin V (Biolegend, USA) and propidium iodide (Biolegend, USA). Doubling time was determined by seeding the cells in 6-well plates at a cell density of 2 × 10^5 cells/ml. The number of vital cells were counted after 24 h, 48 h and 72 h by flow cytometry. The doubling time during the exponential growth phase was subsequently calculated using the formula DT = Tln2/ln(Xe/Xb) (https://www.atcc.org/~/media/PDFs/Culture%20Guides/AnimCellCulture_Guide.ashx), where DT represents the doubling time (in hours), T symbolizes the incubation time (in hours), Xe indicates the cell number at the end of the incubation time, and Xb is the cell number at the beginning of the incubation time.

### Western blotting, RNA isolation and real-time reverse transcriptase polymerase chain reaction (RT-PCR)

Cell lysis and Western blot were carried out as described previously [20]. Twelve percent Expedeon RunBlue SDS protein gels (Biozol Diagnostica Vertrieb GmbH, Germany) were utilized for the run. The primary and secondary antibodies used for Western blot are shown in Table 1.

**Table 1:** List of antibodies

**Target Protein**	**Primary antibody**			
	**Application**		**Company**	**Clone name / catalogue number**
	**Western blot**	**Immunohistochemistry**		
GAPDH	✓		Cell Signaling Technology	14C10
Histone 3	✓		Cell Signaling Technology	96C10
Cleaved PARP	✓		Cell Signaling Technology	Asp214
AHCY	✓	✓	OriGene	TA332593
**Secondary antibody**				
Anti-rabbit HRP-conjugated	✓		GE Healthcare	NA934V
Anti-mouse HRP-conjugated	✓		Agilent	P0447

RNA was isolated using the RNeasy Midi Kit (Qiagen, Germany) adhering to the manufacturer’s recommendations and reverse transcribed to cDNA using TaqMan Reverse Transcription reagents (ThermoFisher Scientific, Germany) on a T3 thermocycler (Biometra GmbH, Germany). Real-time RT-PCR was carried out using TaqMan Gene Expression Assays and TaqMan Gene Expression Master Mix (ThermoFisher Scientific, Germany) following the manufacturer’s protocol on the StepOnePlus Real-Time PCR System (ThermoFisher Scientific, Germany). Beta-2 microglobulin (*B2M*) and succinate dehydrogenase (*SDHA*) genes were used as endogenous controls and the ΔΔC_t_ method [28] was followed for relative mRNA quantification. Details of the respective TaqMan assays used are listed in Table 2.

**Table 2:** TaqMan assays used for gene expression and copy number variation (CNV) analysis

**TaqMan Assays**				
	**Targets**	**Assay ID**	**Type**	**Company**
cDNA target	AHCY	Hs00898137_g1	Gene expression assay	ThermoFisher Scientific
	B2M	Hs00984230_m1	Gene expression assay	ThermoFisher Scientific
	SDHA	Hs00417200_m1	Gene expression assay	ThermoFisher Scientific
gDNA target	AHCY	Hs02422126_cn	CNV assay	ThermoFisher Scientific

### DNA isolation, whole exome sequencing (WES), copy number variation (CNV) assay and OncoScan CNV assay

Genomic DNA was isolated from the cell lines using QIAamp DNA Mini Kit (Qiagen, Germany), following the manufacturer’s instructions. WES was performed on genomic DNA at the Berlin Institute of Health core facility Genomics, Berlin, Germany. Sequencing libraries were prepared with the SureSelect^XT^ Human All Exon v4 library kit (Agilent, Germany) following the manufacturer’s instructions. Cluster Generation was done with the aid of TruSeq PE Cluster Kit v4 (Illumina, USA) and the resulting templates sequenced on an Illumina HiSeq2000 sequencer (at least 150 million reads with a sequencing depth of greater than 160x) using the Illumina HiSeq SBS 250 cycle kit v4.

Data analysis was performed using BWA-MEM [29] to map each whole-exome data set against the reference genome GRCh37. Samblaster [30] was used to mark duplicates. To detect copy number changes, DNA profiles from the respective cell lines were compared (BLUE-1R10 against BLUE-1 K10) with CNVkit [31]. The copy number changes were prioritized according to their log2 fold-change and custom plots of amplified regions created using CNVkit plotting functions.

CNV analysis was performed on genomic DNA from the respective BLUE-1 cell lines, controls and patient samples by applying the TaqMan copy number assay (assay ID: Hs02422126_cn) to the *AHCY* gene on chromosome 20. The assay covers intron 7 and exon 8 on the reference genome GRCh37 and was performed according to the manufacturer’s recommendations. Data analysis was done using the CopyCaller software version 2.1 (ThermoFisher Scientific, Germany).

OncoScan CNV assay Kit was also used to perform copy number analysis according to standard protocols (Affymetrix) [32]. The Chromosome Analysis Suite 4.0 (ChAS) (ThermoFisher, Germany) software was used to visualize, analyze and summarize the chromosomal aberrations, including gains, losses, and loss of heterozygosity (LOH). The non-FFPE analysis work-flow was applied. Criteria for copy number alterations include chromosomal changes encircling at least 20 informative probes, with a minimum size event of at least 100 kb, with median log2Ratio +/− 0.3, and showing a CNN-LOH more than 5 Mb. Individual copy number analysis for each BLUE-1 cell line, as well as, comparative analysis between the three cell lines (BLUE-1, BLUE-1 K10, and BLUE-1R10) was done. In addition, we manually inspected all the aberrations filtered out due to the criteria described above and included only those aberrations showing differences in the B-allele frequency (BAF).

### Clonality studies

B-cell clonality studies were performed to determine the immunoglobulin heavy chain (IGH) rearrangements for BLUE-1, BLUE-1 K10 and BLUE-1R10 using a multiplex PCR method developed within the EuroClonality/BIOMED-2 collaborative study, BMH4-CT98–3936 [33] for all three IGH frame work regions. After PCR on the ProFlex PCR Thermocycler (ThermoFisher Scientific, Germany), gel electrophoresis was performed to check the amplification of PCR products. For single base pair resolution, GeneScan analysis (capillary electrophoresis) of the IGH PCR products was performed with the 3500 series Genetic Analyser (ThermoFisher Scientific, Germany). The sizes of the various PCR products were determined using the GeneMapper 4.0 software (ThermoFisher Scientific, Germany).

### Immunohistochemistry and fluorescence in situ hybridization (FISH) analysis

Immunohistochemistry (IHC) was performed using sections of formalin-fixed paraffin-embedded (FFPE) cell line blocks as described [34]. The primary antibody used in this case was anti-AHCY antibody (Table 1). FISH was performed on sections of formalin-fixed paraffin-embedded (FFPE) cell line blocks as described [35, 36]. This was carried out using orange-labeled *AHCY* gene-specific probes (product name: AHCY-20-OR) and green-labeled chromosome 20-control (centromeric) probes (product name: CHR20–10-GR) (both purchased from Empire Genomics, USA). DakoCytomation Hybridizer (Dako/Agilent, Germany) was used for FISH probe hybridization, while nuclear counterstaining was done with the aid of Dako fluorescence mounting medium containing DAPI (Dako/Agilent, Germany). Visualization and analysis (of at least 50 intact nuclei) were performed with the Zeiss Axio Imager Z1 (Zeiss, Germany) and the Isis imaging software version 5.3.1 (Metasystems, Germany).

### Cytogenetics, metabolomics and global DNA methylation analysis

Profiling of short tandem repeats (STR) for authentication of the cell lines BLUE-1, BLUE-1 K10 and BLUE-1R10 using the StemElite kit (Promega) was performed as previously described [32]. Conventional cytogenetic analysis was performed as reported [37] and the karyotypes were described according to ISCN guidelines (2013).

The intermediates of methionine and polyamine metabolism in BLUE-1, BLUE-1 K12 and BLUE-1R12 cell extracts were measured by liquid chromatography-tandem mass spectrometry following an established protocol [38]. Genome-wide DNA methylation analysis was done on BLUE-1, BLUE-1 K10 and BLUE-1R10 using the Infinium MethylationEPIC BeadChip (Illumina, USA) as described [39]. Copy number plots were generated from the raw output data (*.idat* files) using the ‘conumee’ R package in Bioconductor [39, 40].

### Statistical analysis

Statistical analysis was done using the GraphPad Prism 5 software (GraphPad Software, California, USA). Statistical significance was evaluated using the Mann-Whitney U test (two-tailed) for pairwise comparisons, and one-way ANOVA with the Tukey post-hoc test for group comparisons. *p* values less than 0.05 were considered significant.

## Results

### Generation and characterization of a DZNep-resistant cell line clone

We generated a DZNep-resistant clone by subjecting the DZNep-sensitive Burkitt lymphoma cell line BLUE-1 [20] to progressively increasing concentrations of DZNep for up to 7 months (Fig. 1a and Methods section). Analyses were performed with the resistant BLUE-1 subclones (BLUE-1R10 or BLUE-1R12) and their respective controls. To analyze the response of the generated DZNep-resistant clone to DZNep, we treated the clone and its corresponding control with 5 μM DZNep for 72 h and then measured the percentage of apoptotic cells. The control cell line, BLUE-1 K10, exhibited strong apoptosis with about 50% apoptotic cells in comparison to the resistant BLUE-1R10 clone, which displayed only about 10% apoptotic cells following treatment with DZNep (Fig. 1b). Furthermore, Western blot analysis performed on total protein lysate obtained after treatment of both cell lines with 2 μM and 5 μM DZNep revealed an increase in the expression of cleaved PARP, indicating apoptosis in the DZNep-treated cells of BLUE-1 K10 in relation to BLUE-1R10 (Fig. 1b). The doubling time of the DZNep-resistant clone BLUE-1R10 was also compared with that of the corresponding control BLUE-1 K10 and the parent cell line BLUE-1. This revealed that both BLUE-1R10 and BLUE-1 K10 had shorter doubling times than BLUE-1 (Fig. 1c).

To determine the identity of the generated clone, we explored the STR profile and determined the clonality of BLUE-1R10. The results were then compared with those of its corresponding control BLUE-1 K10 and the parent cell line BLUE-1. The STR profile analysis for the three cell lines when compared with the DSMZ STR profiling database revealed an STR profile of a BLUE-1 cell line, confirming their authenticity. Furthermore, the IGH chain gene rearrangement patterns for the three cell lines were identical (Additional file 1: Figure S1). We also performed genomic characterization of the cell lines using conventional cytogenetics and copy number analysis by OncoScan CNV assay. Only minor differences in the karyotype were observed upon analysis of the three cell lines (Additional file 2: Figure S2). Upon further examination the copy number data, we detected genomic aberrations exclusive to each of the three BLUE-1 cell lines (Additional file 5: Table S1). We detected three abnormalities only present in BLUE-1R10 as compared to the parental BLUE-1 and BLUE-1 K10 cell lines. These include one loss in 4q12q12, one high copy gain in 6q14.3q14.3, and one LOH in 20p12.2p13. Moreover, we observed aberrations solely in the parental BLUE-1 cell line, including gains in 6p22.1p25.3, 19p13.11p13.3, and 19p13.11q13.43, which may have been lost due to prolonged culture conditions. We also distinguished common copy number aberrations in BLUE-1 and BLUE-1R10 cell lines, but not shown in the BLUE-1 K10 (Additional file 5: Table S1).

### Identification of biomarkers for resistance to DZNep

**Fig. 2:**
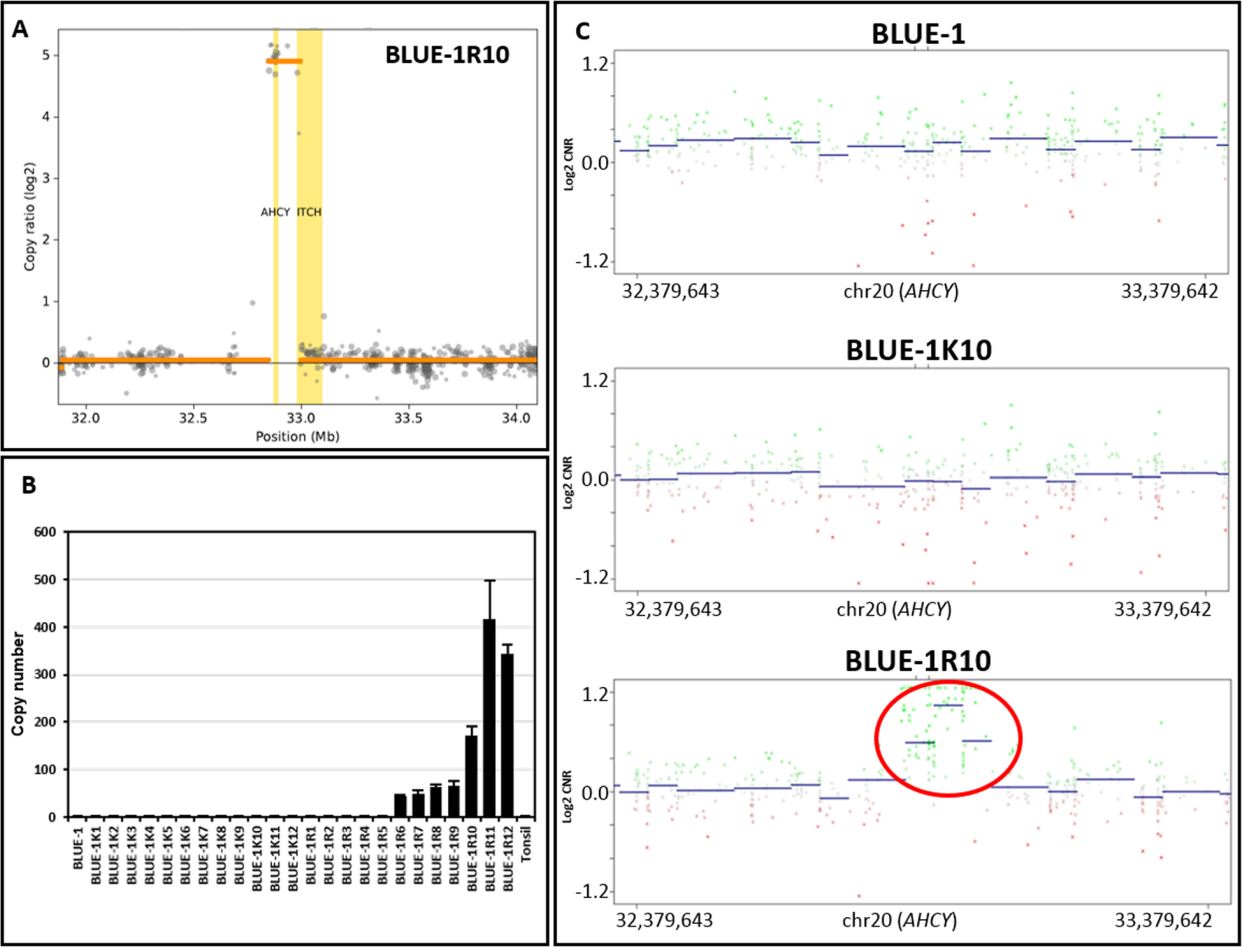
*AHCY* copy number gain in the DZNep-resistant clone. **a** Log2 copy ratio plot of copy number variation regions in BLUE-1R10 in relation to BLUE-1 K10. The gray dots represent copy ratio values across different bins, the orange line shows segments and the yellow vertical lines indicate the respective genes. **b** Evolution of *AHCY* copy number in the resistant clone. Genomic DNA from the cell lines was subjected to the TaqMan copy number assay (ID: Hs02422126_cn). The real-time PCR read-out and copy number was analyzed with the CopyCaller software. A human tonsil DNA sample was used as a calibrator for the TaqMan copy number assay. **c** CNV plots calculated from global DNA methylation array data. Chromosome 20 locus on BLUE-1, BLUE-K10 and BLUE-1R10 was analyzed for variations in *AHCY* copy number. The y-axis represent the log2 copy number ratio (CNR). Amplifications represent positive deviations from the baseline while losses indicate negative deviations from the baseline (0.0). Encircled in red, shows *AHCY* copy number gain in BLUE-1R10

To probe the underlying mechanism of resistance of BLUE-1R10 to DZNep, we performed WES of genomic DNA obtained from BLUE-1R10 and its corresponding control BLUE-1 K10. Additional copy number variant analysis was performed on the WES data using the software tool CNVkit. This analysis identified a small focal, approximately 30-fold copy number gain in the region spanning the *AHCY* gene and the proximal part of the *ITCH* gene on chromosome 20 (Fig. 2a). Upon manual inspection of this specific genomic region in the OncoScan data, we confirmed the presence of this high copy number gain including the *AHCY* gene (Additional file 3: Figure S3). It was not called in the initial analysis due to applied detection criteria for the analysis which filtered the amplicon out due to its small genomic size and low marker content (see material and method sections for more details).

We validated this *AHCY* gain on the DNA level using two methods. First, we used the TaqMan Copy Number Assay to analyze DNA obtained from cryostocks collected at the various time-points during the generation of the resistant clone, including BLUE-1 as the reference cell line. We noted a clear copy number gain in *AHCY* beginning with BLUE-1R6 (preserved after almost 5 months of treatment with DZNep) and increasing thereafter (Fig. 2b). The *AHCY* copy number gain was further confirmed by a CNV analysis using global DNA-methylation data from BLUE-1R10, and comparing it with data from BLUE-1 K10 and BLUE-1. Here, the gain in *AHCY* copy number on chromosome 20 was also obvious in the resistant clone in comparison to the respective control and the parent cell line (Fig. 2c).

### *AHCY* copy number gain at the chromosomal and transcriptional level

**Fig. 3:**
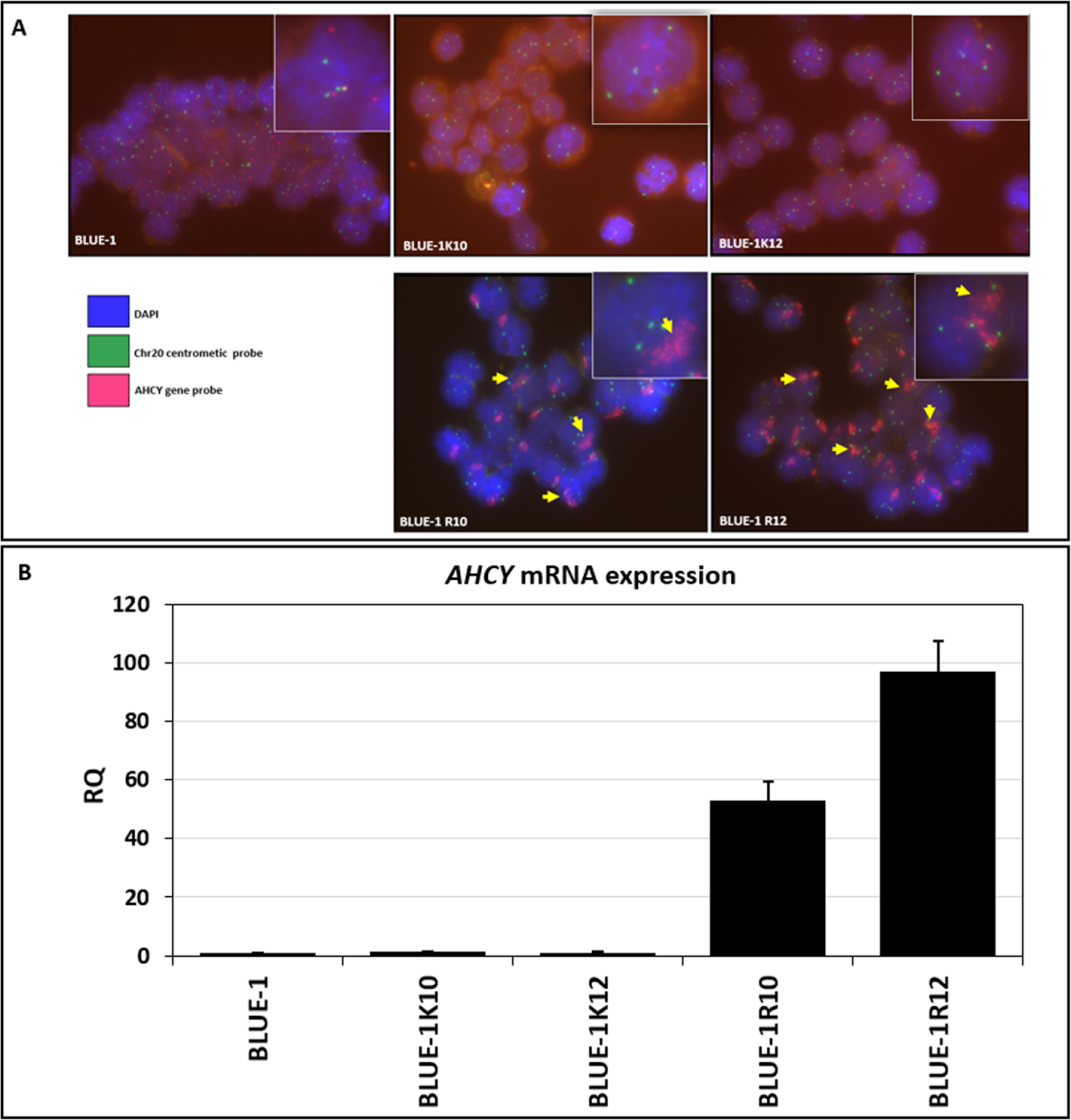
*AHCY* copy number gain in the DZNep-resistant clone: chromosomal and transcriptional validation. **a** FISH analysis using *AHCY* target-specific probes in the resistant clone and its control. Yellow arrows show in pink color a cluster-type amplification of *AHCY*. Green dots represent the centromere of chromosome 20. **b** Transcriptional expression of *AHCY*. cDNA was synthesized from the RNA of all cell lines. Relative gene expression (shown on the y-axis) was quantified using the *AHCY* gene expression assay, with B2M and SDHA used as an endogenous control. RQ is shown as mean plus SD of triplicate measurements

On the chromosomal level, we confirmed *AHCY* amplification in BLUE-1R10 and BLUE-1R12, by performing a FISH study using target-specific probes for *AHCY* on chromosome 20q11.22. Our FISH data (Fig. 3a) revealed cluster-type amplification and large *AHCY*-gene clusters in form of dense clouds of labeled regions suggestive for hsr-regions in cells of BLUE-1R10, when compared with its control BLUE-1 K10 and BLUE-1.

To ascertain the expression of AHCY, we performed a real-time PCR using the TaqMan gene expression assay on cDNA from the resistant clone, the respective controls and the parent cell line BLUE-1. The results display overexpression of AHCY in the resistant clone BLUE-1R10 and a further increase in BLUE-1R12 (Fig. 3b).

### *AHCY* gain at the protein level and metabolomics studies

**Fig. 4:**
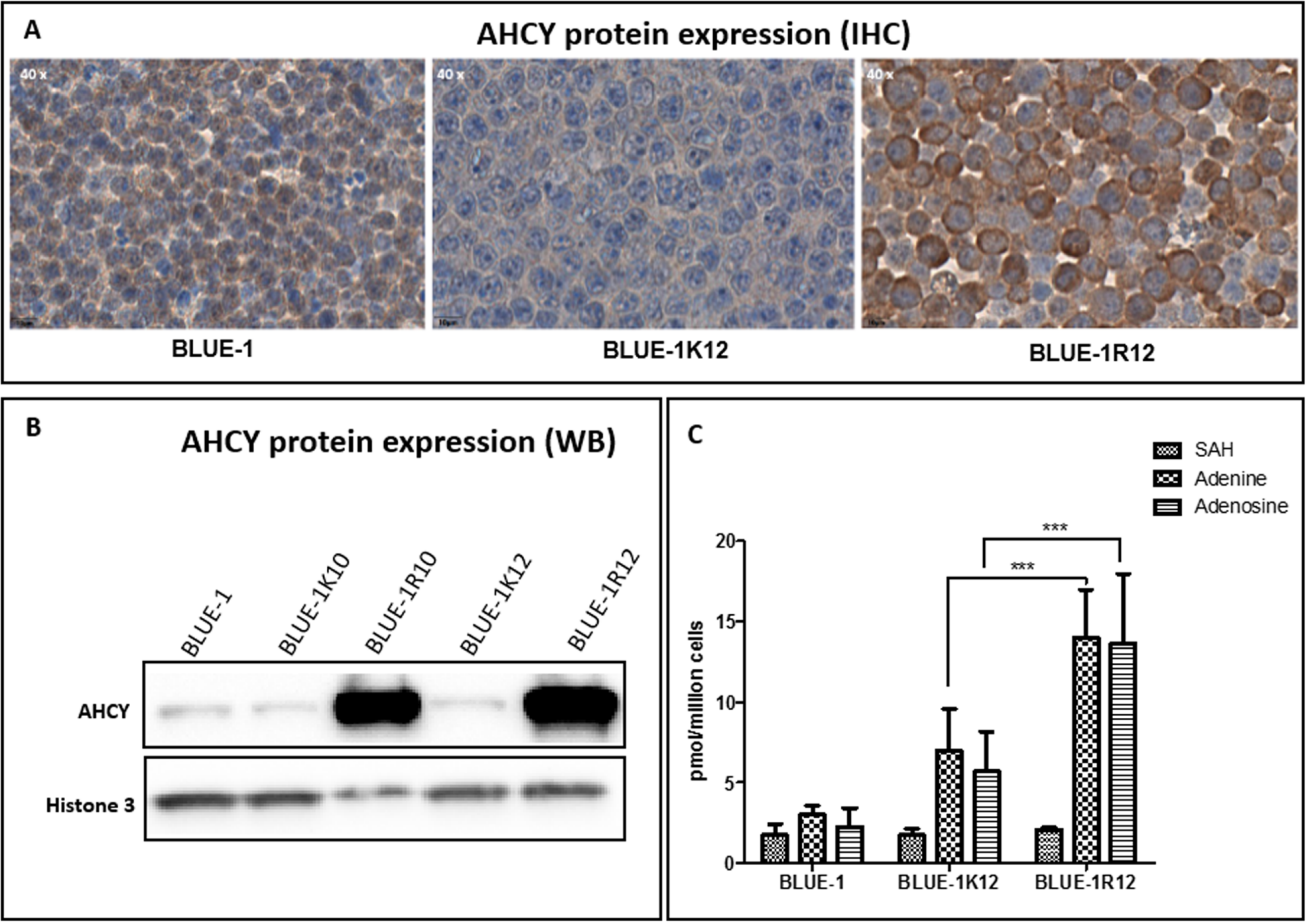
Translational validation of AHCY gain in the resistant clone, and metabolomics analysis. **a** IHC results from BLUE-1, BLUE-1 K12 and BLUE-1R12 cell lines. Sections from FFPE cell line blocks were stained with anti-AHCY antibody. **b** Validation of AHCY overexpression at the protein level using Western blot. Whole cell protein lysates from the cell lines were analyzed using Western blot. Histone 3 was used as a loading control for the blot. The full-length blot is presented in Additional file 7: Fig. S6. FUSION-CAP Software was used for Western blot image analysis. **c** Quantification of S-adenosyl methionine, adenine and adenosine in BLUE-1, BLUE-1 K12 and BLUE-1R12. Values are shown as mean plus SD from six replicate measurements

AHCY expression at the protein level was determined using two different methods, IHC and Western blot. The IHC results show increased AHCY expression in the resistant clone in comparison to its control and the parent cell line (Fig. 4a). The Western blot result (Fig. 4b) confirmed that AHCY is overexpressed in BLUE-1R10, with a higher expression in BLUE-1R12. The respective controls and parent BLUE-1 cell line had a similar level of AHCY expressed.

To understand the dynamics of the concentrations of methionine intermediates in the resistant clone, its control and the parent cell line, we employed a high performance liquid chromatography-tandem mass spectrometry method. We notice a similar distribution pattern for S-adenosyl-L-homocysteine (SAH), adenine and adenosine in BLUE-1 and the control cell line BLUE-1 K12. The resistant clone (BLUE-1R12), however, displayed a significant increase (*p* < 0.05) in the level of both adenine and adenosine in comparison to its control BLUE-1 K12 (Fig. 4c).

### *AHCY* copy number gain in primary lymphoma samples

We examined the frequency of *AHCY* copy number gains in a small series of B-cell lymphomas by examining 12 primary lymphoma samples consisting of Burkitt lymphoma, diffuse large B-cell lymphoma, follicular lymphoma, primary mediastinal B-cell lymphoma and anaplastic large cell lymphoma. We performed the TaqMan CNV assay using genomic DNA isolated from these samples. Subsequent analysis of the *AHCY* copy number with the CopyCaller software revealed a predicted *AHCY* copy number of 2 for all but one sample, which had a predicted copy number of 1 (Additional file 4: Figure S4). In addition, we checked the frequency of *AHCY* copy number alterations in B-cell lymphoma by analyzing published genomic data from 1295 B-cell lymphoma samples curated from 5 different studies on the cBio Cancer Genomics Portal [41, 42] but no *AHCY* genomic alterations were detected.

## Discussion

Acquired resistance to small molecule inhibitors used in cancer treatment remains a huge problem in cancer therapy. Although many cancer types respond to initial therapy, there is the uncertainty of resistance arising against the utilized drug [43]. Various molecular mechanisms are involved in the resistance of tumor cells to therapy [44–46]. DZNep - an indirect EZH2 inhibitor - is known to be efficacious against many different types of cancer and, hence, may make its way into clinical trials. Already, genetic determinants of sensitivity to DZNep-mediated apoptosis have been described for gastric cancer and multiple myeloma tumor cells respectively [47, 48]. In this study, we aimed to investigate the molecular mechanism underlying acquired resistance to DZNep in B-cell lymphoma, and to identify biomarkers predictive of the therapeutic success of EZH2 inhibition with DZNep. To achieve this, we generated and investigated a DZNep-resistant clone. The continued resistance of the clone (BLUE-1R10) to DZNep following treatment with DZNep as well as upon cultivation in a DZNep-free medium implies that a permanent change has occurred on the genomic level in this clone. We analyzed the proliferation rate of BLUE-1R10, comparing its doubling time with that of BLUE-1 K10 and BLUE-1. The shorter doubling time observed in the resistant clone and its control in relation to the parent cell line is in contrast to a previous report of an increased doubling time in two colon cancer cell lines that acquired drug resistance upon prolonged cultivation with irinotecan [49]. Since the reduction in doubling time was also observed in the control cell line BLUE-1 K10, the increased growth rate of both BLUE-1R10 and BLUE-1 K10 cannot be attributed to the effect of DZNep on the cells. Perhaps, some changes in genes responsible for cell cycle regulation and proliferation could have occurred during the long-term cultivation of the clone and its control. This is not surprising because, we already know the extent of genetic and transcriptional heterogeneity that occur in cell lines during evolution [50]. Besides, in other cell types such as human embryonic stem cells, the effect of prolonged cultivation on the proliferative capacity is evident as an increase in the proliferative capacity of these cells [51].

It was crucial to confirm the identity of the DZNep-resistant clone to ensure that it certainly originated from the parent cell line BLUE-1. To achieve this, the STR profile and clonality of BLUE-1R10 was explored together with that of BLUE-1 K10 and BLUE-1. The identical STR profile and IGH chain gene rearrangement patterns indicate that both cell lines indeed originate from the same parent. Nevertheless, the differences observed between BLUE-1, BLUE-1 K10 and BLUE-1R10 upon genomic interrogation using the OncoScan CNV assay could suggest a divergent genomic evolution between BLUE-1 K10 and BLUE-1R10 cell lines in culture.

Since AHCY is a direct target of DZNep-mediated EZH2 inhibition, we focused on validating the identified *AHCY* gain in the resistant clone. Using the TaqMan Copy Number Assay, which measures gene copy numbers by incorporating the TaqMan copy number assay with the TaqMan copy number reference assay in a single real-time PCR run, validation of *AHCY* amplification was achieved on the DNA level. The progressive increase in the *AHCY* copy number observed from BLUE-1R6 confirms that the gain in copy number is continual in the DZNep-resistant clone. It is noteworthy that at the point of DZNep withdrawal from the clone (from BLUE-1R9 to BLUE-1R10), there was an almost two-fold increase in the *AHCY* copy number. This copy number gain doubled following continuous cultivation of the clone in a DZNep-free medium (from BLUE-1R10 to BLUE-1R11 and BLUE-1R12). This implies that the *AHCY* copy number gain, once initiated, does not require DZNep pressure to persist. Although this copy number event may reflect a sort of genomic compensation in the resistant clone due to prolonged AHCY inhibition, it is unlikely that this genomic aberration would decrease to baseline levels upon prolonged withdrawal of DZNep pressure.

Moreover, the FISH data which revealed that the *AHCY* amplification was more pronounced in BLUE-1R12 in comparison to BLUE-1R10 further affirms the persistence of *AHCY* copy number gain. The chromosome 20 polysomy observed in the cells of each cell line may reflect the level of genomic instability usually occurring in cancer cells [52, 53]. The overexpression of AHCY on the transcriptional and protein level in the DZNep-resistant clone is consistent with the knowledge of drug resistance in cancer stemming from alterations in the drug target, particularly, alterations involving modified enzyme expression [46, 54]. Alterations in the apoptotic machinery did not appear to be involved in the resistance of the resistant clone to apoptosis based on its RNA expression profile.

The results of our metabolomics studies which revealed an analogous distribution for SAH, adenine and adenosine in both BLUE-1 and BLUE-1 K12 signifies that following long-term culture of BLUE-1, there is no peculiar alteration of the balance exerted by these intermediates within the cell. The increase in adenine and adenosine levels of the resistant clone (BLUE-1R12) when compared to BLUE-1 K12 is in line with the increased expression of AHCY, which catalyzes the hydrolysis of SAH to adenosine and L-homocysteine.

Previous works have linked copy number gains on chromosome 20q with the pathogenesis of tumors such as colon cancer [55], colorectal cancer [56], and cervical cancer [57], with significant gains of the *AHCY* gene among others. However, little is known about the role of *AHCY* copy number gain in driving B-cell lymphomas. For this reason, we analyzed the *AHCY* copy number of 12 primary lymphoma samples using the TaqMan CNV assay. The absence of *AHCY* copy number gain in the patient samples analyzed implies that it is unlikely that *AHCY* copy number gains play a driving role in B-cell lymphoma pathogenesis. In addition, the absence of AHCY copy number alterations from in silico studies signifies that alterations in *AHCY* are rare in primary B-cell lymphoma however, in solid tumors, the frequency of *AHCY* amplification can be as high as 20% [41, 42].

## Conclusion

Acquired drug resistance poses a great challenge in cancer therapy. It is important to recognize mechanisms of resistance for novel drugs about to enter clinical trials so that one can monitor for early signs of development of drug resistance. DZNep is a promising epigenetic drug that is in the pre-clinical phase of clinical approval, but has shown promising effects for selected cancer patients. We show that copy number gain of *AHCY* is one possible mechanism of acquired resistance to DZNep-mediated apoptosis and propose AHCY as a potential biomarker to stratify patients during the use of DZNep. Although AHCY alterations are rare in primary B-cell lymphomas, it may still be important to screen for modifications of this gene in patients prior to the initiation of EZH2 based therapy with DZNep. These findings might be valuable in predicting patients, who will benefit from EZH2 inhibition using DZNep once it progresses into clinical studies.

## Supplementary information

**Additional file 1: Figure S1.** Confirmation of the identical B-cell clonality of BLUE-1K10 and BLUE-1R10.

**Additional file 2: Figure S2.** Karyotypes of the cell lines BLUE-1, BLUE-1R10 and BLUE-1K10.

**Additional file 3: Figure S3.** OncoScan Copy number analysis in the of BLUE-1 cell lines.

**Additional file 4: Figure S4.** Analysis of *AHCY* copy number in primary lymphoma samples.

**Additional file 5: Table S1.** Comparative copy number analysis in the BLUE-1 cell lines as determined by OncoScan assay.

**Additional file 6: Figure S5.** Full-length blot for Western blot image presented in Fig. 1.

**Additional file 7: Figure S6.** Full-length blot for Western blot image presented in Fig. 4.

## Data Availability

The datasets supporting the conclusions of this article are included within the article and its additional files.

## Abbreviations

AHCY: S-adenosyl-L-homocysteine hydrolase
B2M: Beta 2 microglobulin
BAF: B-allele frequency
ChAS: Chromosome analysis suite
CN-LOH: Copy neutral loss of heterozygosity
CNR: Copy number ratio
CNV: Copy number variation
DZNep: 3-Deazaneplanocin A
DSMZ: German Collection of Microorganisms and Cell Cultures
EZH2: Enhancer of zeste homolog 2
FFPE: Formalin-fixed paraffin-embedded
FISH: Fluorescence in situ hybridization
H3K27me3: Lysine 27 trimethylation on histone 3
IGH: Immunoglobulin heavy chain
IHC: Immunohistochemistry
LOH: Loss of heterozygosity
RT-PCR: Reverse transcriptase polymerase chain reaction
SAH: S-adenosyl-L-homocysteine
SDHA: Succinate dehydrogenase
STR: Short tandem repeat
WES: Whole exome sequencing
