# Supplementary material for: Acquired resistance to DZNep-mediated apoptosis is associated with copy number gains of AHCY in a B-cell lymphoma model
Source: BMC Cancer. 2020 May 14;20:427. doi: 10.1186/s12885-020-06937-8 (PMC7227222; doi:10.1186/s12885-020-06937-8)
Supplement: Supplementary file 1 — Additional file 1: Figure S1. Confirmation of the identical B-cell clonality of BLUE-1K10 and BLUE-1R10. [file 12885_2020_6937_MOESM1_ESM.pdf]

Additional file 1.

Figure S1. Confirmation of the identical B-cell clonality of BLUE-1K10 and BLUE-1R10.

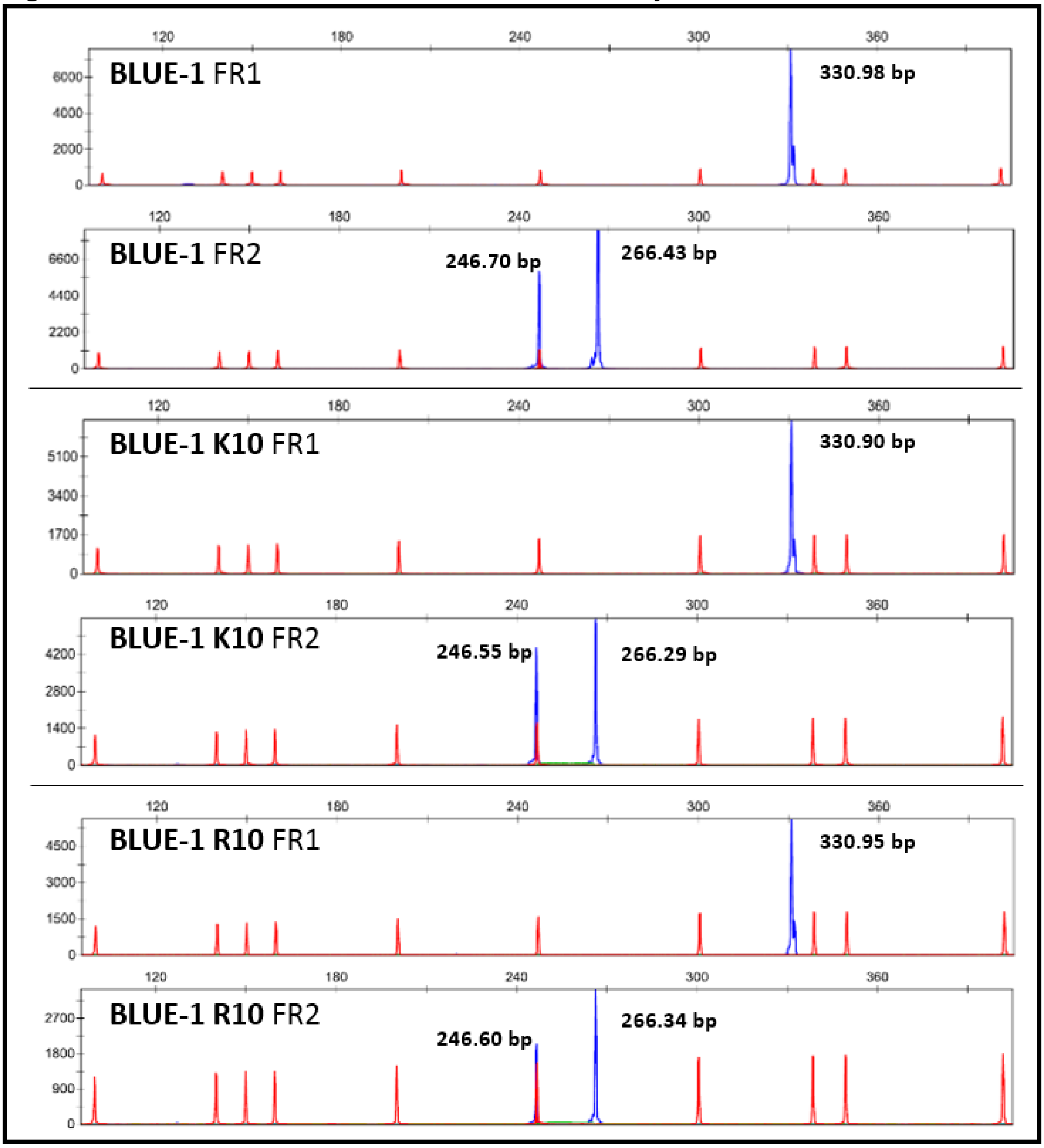

Multiplex IGH PCR was performed on DNA isolated from the respective cell lines using specific primer sets for framework (FR) 1 and FR2 regions of the variable (V) segment, and consensus primers for the joining (J) segment of IGH gene. The figure shows monoallelic rearrangements (one dominant peak) of FR1 sequences and biallelic rearrangements (two dominant peaks) of the FR2 sequences in GeneScan analysis. Red peaks indicate size markers.
