# Supplementary material for: Acquired resistance to DZNep-mediated apoptosis is associated with copy number gains of AHCY in a B-cell lymphoma model
Source: BMC Cancer. 2020 May 14;20:427. doi: 10.1186/s12885-020-06937-8 (PMC7227222; doi:10.1186/s12885-020-06937-8)
Supplement: Supplementary file 2 — Additional file 2: Figure S2. Karyotypes of the cell lines BLUE-1, BLUE-1R10 and BLUE-1K10. [file 12885_2020_6937_MOESM2_ESM.pdf]

Additional file 2.

Figure S2. Karyotypes of the cell lines BLUE-1, BLUE-1R10 and BLUE-1K10.

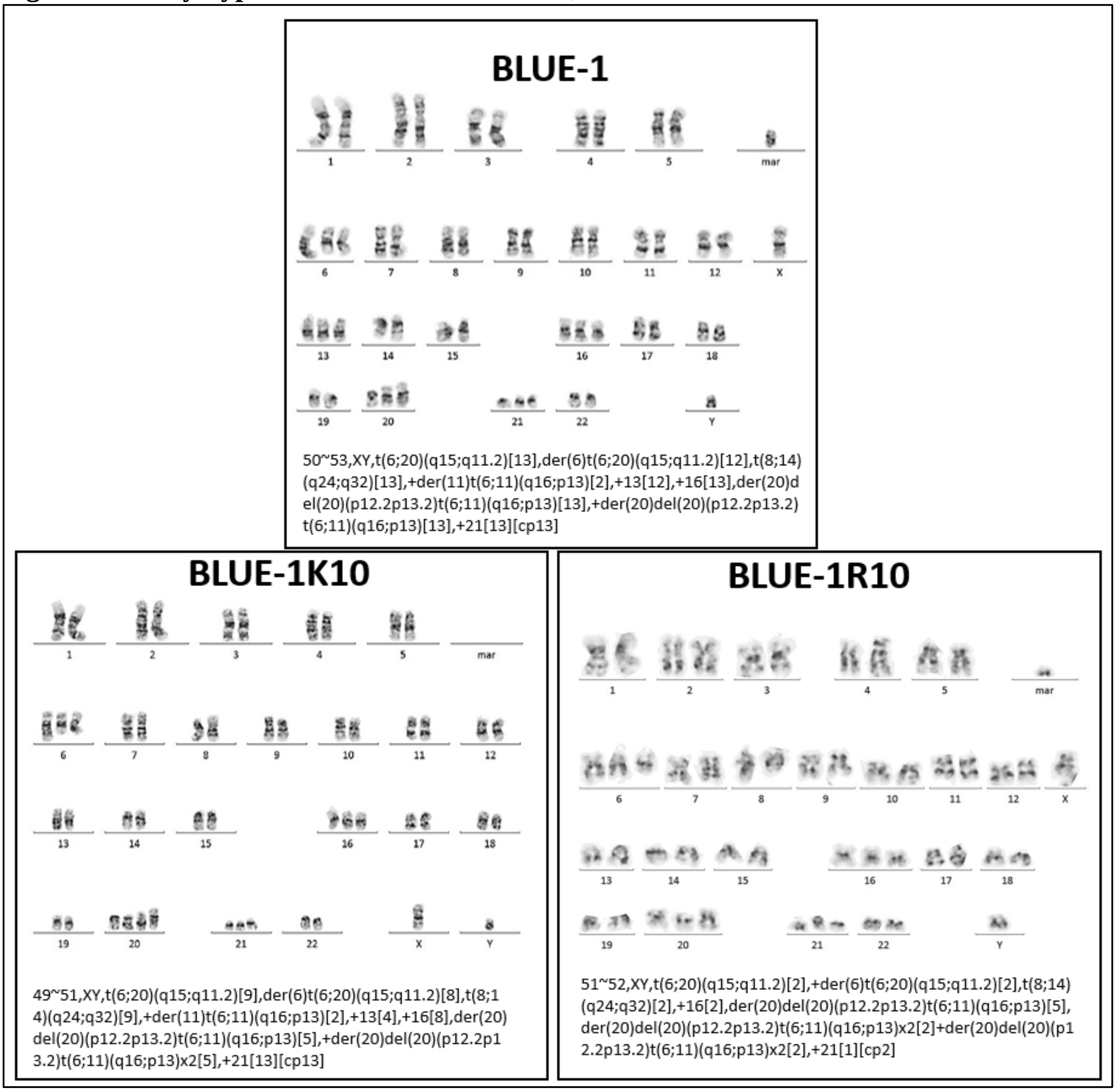

'mar' means marker chromosome (when the origin of the chromosome is unknown). '+' shows that there are more than 2 copies for the respective chromosome. 'cp' used at the end of the formula indicates that this is a composite karyotype and the number of metaphases are included with each aberration in square brackets '[ ]'. The international nomenclature (ISCN 2013) was used to write the karyotype.
