# Supplementary material for: Acquired resistance to DZNep-mediated apoptosis is associated with copy number gains of AHCY in a B-cell lymphoma model
Source: BMC Cancer. 2020 May 14;20:427. doi: 10.1186/s12885-020-06937-8 (PMC7227222; doi:10.1186/s12885-020-06937-8)
Supplement: Supplementary file 3 — Additional file 3: Figure S3. OncoScan Copy number analysis in the of BLUE-1 cell lines. [file 12885_2020_6937_MOESM3_ESM.pdf]

Additional file 3.

Figure S3. OncoScan Copy number analysis in the of BLUE-1 cell lines.

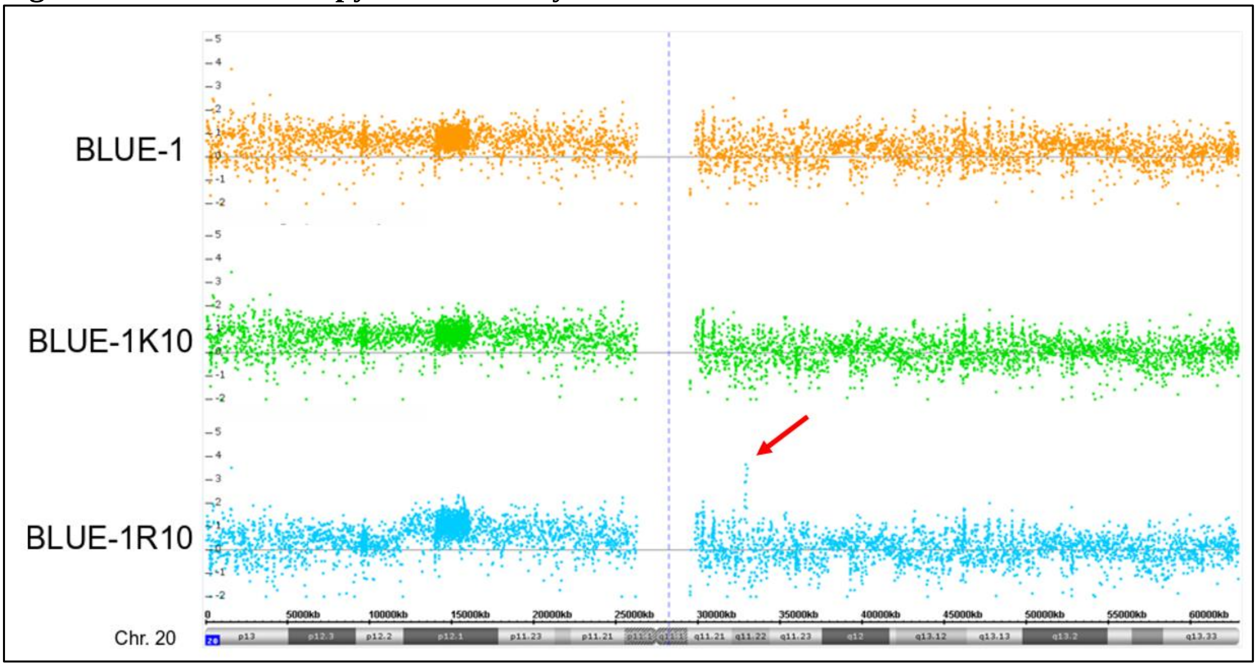

Displayed are the log2 ratios for the three BLUE-1 cell lines on chromosome 20. The copy number analysis for the *AHCY* gene located on chromosomal region 20q11.21 showed a high copy number gain in BLUE-1R10 cell line (highlighted with the red arrow) as compared to BLUE-1, as well as BLUE-1K10.
