# Supplementary material for: Acquired resistance to DZNep-mediated apoptosis is associated with copy number gains of AHCY in a B-cell lymphoma model
Source: BMC Cancer. 2020 May 14;20:427. doi: 10.1186/s12885-020-06937-8 (PMC7227222; doi:10.1186/s12885-020-06937-8)
Supplement: Supplementary file 4 — Additional file 4: Figure S4. Analysis of AHCY copy number in primary lymphoma samples. [file 12885_2020_6937_MOESM4_ESM.pdf]

Additional file 4.
Figure S4. Analysis of *AHCY* copy number in primary lymphoma samples.

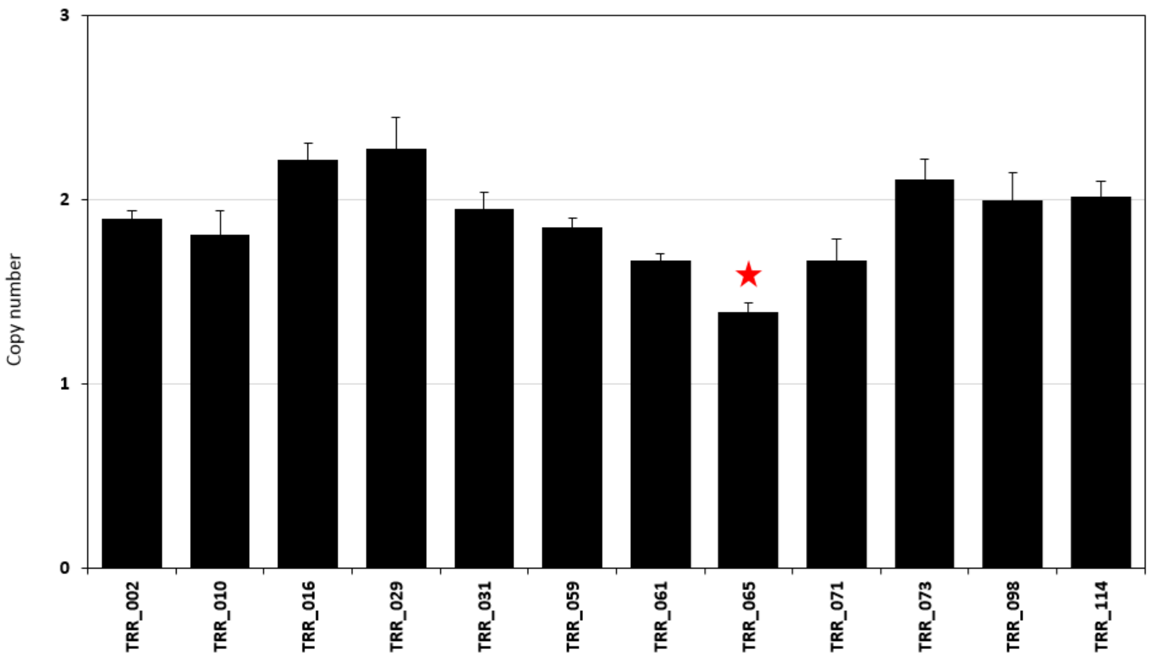

Genomic DNA from the individual samples were used for the validation of AHCY CNV using TaqMan CNV assay (ID: Hs02422126_cn). The real-time PCR read-out and copy number was analyzed with the CopyCaller software. A primary tonsil DNA sample (TRR_098) was used as a calibrator for the analysis. The red highlighted sample shows the sample with a predicted copy number of 1.
