# Supplementary material for: Acquired resistance to DZNep-mediated apoptosis is associated with copy number gains of AHCY in a B-cell lymphoma model
Source: BMC Cancer. 2020 May 14;20:427. doi: 10.1186/s12885-020-06937-8 (PMC7227222; doi:10.1186/s12885-020-06937-8)
Supplement: Supplementary file 5 — Additional file 5: Table S1. Comparative copy number analysis in the BLUE-1 cell lines as determined by OncoScan assay. [file 12885_2020_6937_MOESM5_ESM.pdf]

Additional file 5.
Table S1. Comparative copy number analysis in the BLUE-1 cell lines as determined by OncoScan assay.

Aberration	Genomic region (hg19)	Size (kbp)	Ensembl Gene Count	Cell lines
Loss	chr4:53960195-54283313	323.118	4	BLUE-1-R10
Gain	chr6:204908-28013410	27808.502	396	BLUE-1
High copy gain	chr6:86373374-87809402	1436.028	9	BLUE-1-R10
Gain	chr13:19084822-115103150	96018.328	968	BLUE-1 & BLUE-1K10*
Gain	chr16:8947035-11210415	2263.38	176	BLUE-1 & BLUE-1R10
Gain	chr16:11229588-23143876	11914.288	787	BLUE-1 & BLUE-1R10
Gain	chr16:23156450-90158005	67001.555	4049	BLUE-1 & BLUE-1R10
Gain	chr19:18306750-59093239	40786.489	1597	BLUE-1
Gain	chr19:247231-18101830	17854.599	866	BLUE-1
Gain	chr19:27754572-59093239	31338.667	1419	BLUE-1 & BLUE-1R10
CN-LOH	chr20:69093-11891608	11822.515	213	BLUE-1-R10

*For the BLUE-1K10, the software called the aberrations as two events

CN-LOH: copy neutral loss of heterozigosity
