# Supplementary figures and images for: Acquired resistance to DZNep-mediated apoptosis is associated with copy number gains of AHCY in a B-cell lymphoma model
Source: BMC Cancer. 2020 May 14;20:427. doi: 10.1186/s12885-020-06937-8 (PMC7227222; doi:10.1186/s12885-020-06937-8)

Additional file 6.

Figure S5. Full-length blot for Western blot image presented in figure 1.

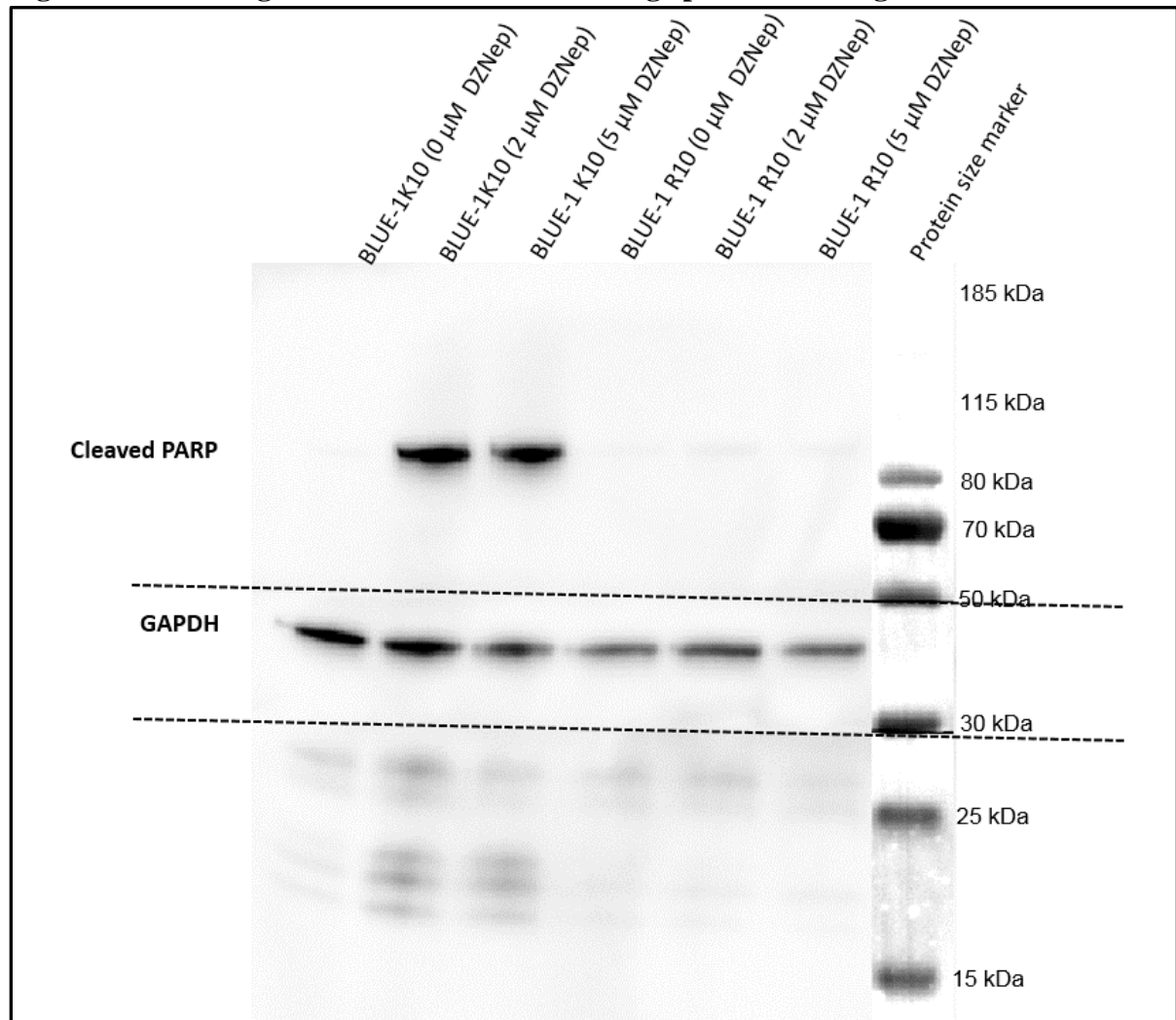

Supplement: Supplementary file 6 — Additional file 6: Figure S5. Full-length blot for Western blot image presented in Fig. 1. [file 12885_2020_6937_MOESM6_ESM.pdf]

**Additional file 7.**

**Figure S6. Full-length blot for Western blot image presented in figure 4.**

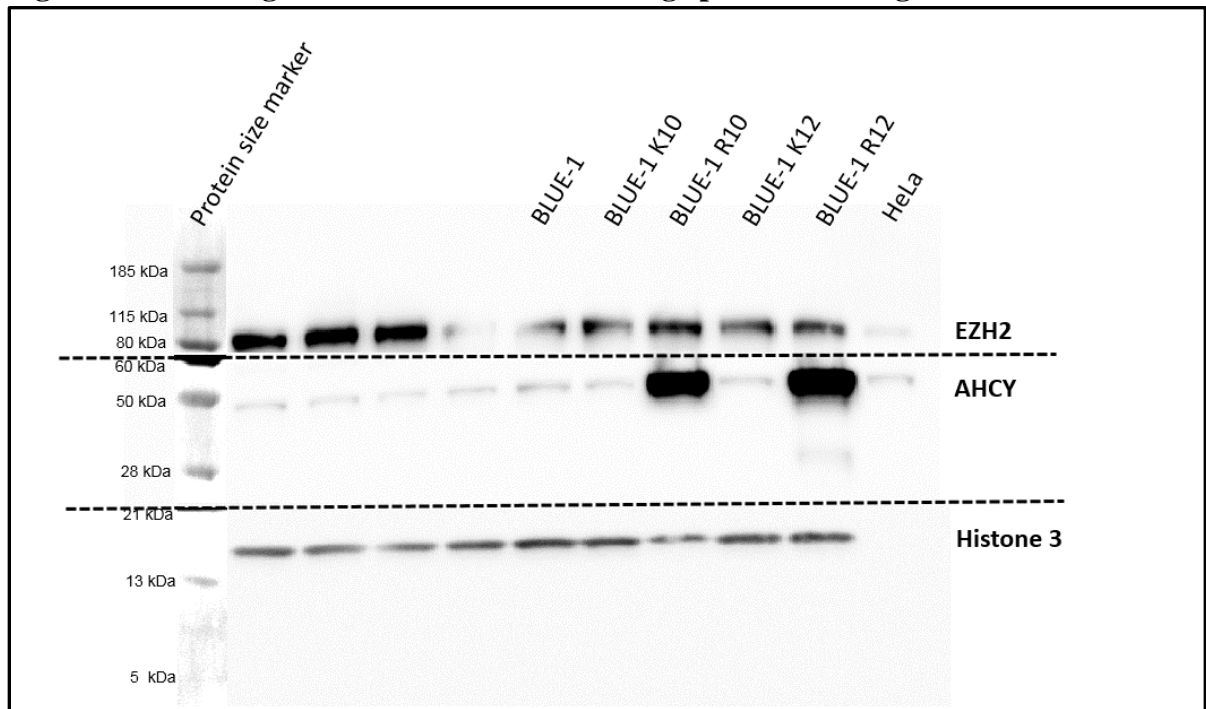

Supplement: Supplementary file 7 — Additional file 7: Figure S6. Full-length blot for Western blot image presented in Fig. 4. [file 12885_2020_6937_MOESM7_ESM.pdf]
